# Safe Medication in Nursing Home Residents Through the Development and Evaluation of an Intervention (SAME): Protocol for a Fully Integrated Mixed Methods Study With a Cocreative Approach

**DOI:** 10.2196/43538

**Published:** 2023-03-31

**Authors:** Marie Haase Juhl, Ann Lykkegaard Soerensen, Jette Kolding Kristensen, Søren Paaske Johnsen, Anne Estrup Olesen

**Affiliations:** 1 Department of Clinical Medicine Aalborg University Aalborg Denmark; 2 Department of Clinical Pharmacology Aalborg University Hospital Aalborg Denmark; 3 University College of Northern Denmark Aalborg Denmark; 4 Research Unit for General Practice in Aalborg Aalborg University Aalborg Denmark; 5 Danish Center for Health Services Research Department of Clinical Medicine Aalborg University Aalborg Denmark

**Keywords:** protocols and guidelines, medication safety, cocreation, user involvement, health and safety within primary care, research in nursing home care, mixed methods

## Abstract

**Background:**

Medication safety is increasingly challenging patient safety in growing aging populations. Developing positive patient safety cultures is acknowledged as a primary goal to improve patient safety, but evidence on the interventions to do so is inconclusive. Nursing home residents are often cognitively and physically impaired and are therefore highly reliant on frontline health care providers. Thus, interventions to improve medication safety of nursing home residents through patient safety culture among providers are needed. Using cocreative partnerships, integrating knowledge of residents and their relatives, and ensuring managerial support could be beneficial.

**Objective:**

The primary aim of the Safe Medication of Nursing Home Residents Through Development and Evaluation of an Intervention (SAME) study is to improve medication safety for nursing home residents through developing an intervention by gaining experiential knowledge of patient safety culture in cocreative partnerships, integrating knowledge of residents and their relatives, and ensuring managerial support.

**Methods:**

The fully integrated mixed method study will be conducted using an integrated knowledge translation approach. Patient safety culture within nursing homes will first be explored through qualitative focus groups (stage 1) including nursing home residents, their relatives, and frontline health care providers. This will inform the development of an intervention in a multidisciplinary panel (stage 2) including cocreators representing the medication management process across the health care system. Evaluation of the intervention will be done in a randomized controlled trial set at nursing homes (stage 3). The primary outcome will be changes in the mean scale score of an adapted version of the Danish “Safety Attitudes Questionnaire” (SAQ-DK) for use in nursing homes. Patient safety–related outcomes will be collected through Danish health registers to assess safety issues and effects, including medication, contacts to health care, diagnoses, and mortality. Finally, a mixed methods analysis on patient safety culture in nursing homes will be done (stage 4), integrating qualitative data (stage 1) and quantitative data (stage 3) to comprehensively understand patient safety culture as a key to medication safety.

**Results:**

The SAME study is ongoing. Focus groups were carried out from April 2021 to September 2021 and the workshop in September 2021. Baseline SAQ-DK data were collected in January 2022 with expected follow-up in January 2023. Final data analysis is expected in spring 2024.

**Conclusions:**

The SAME study will help not only to generate evidence on interventions to improve medication safety of nursing home residents through patient safety culture but also to give insight into possible impacts of using cocreativity to guide the development. Thus, findings will address multiple gaps in evidence to guide future patient safety improvement efforts within primary care settings of political and scientific scope.

**Trial Registration:**

ClinicalTrials.gov NCT04990986; https://clinicaltrials.gov/ct2/show/NCT04990986

**International Registered Report Identifier (IRRID):**

DERR1-10.2196/43538

## Introduction

### Background

#### Medication Safety in Nursing Homes

Despite years of focus, medication safety is still troubling patient safety globally, with increasing demands on health care systems challenged by aging populations [1]. Physical and social environments constitute system factors that are thought to be the main factors influencing health of older adults [2]. Medication errors and unsafe medication practices are the leading causes of avoidable harm in health care, playing a key role in jeopardizing patient safety. Nursing home residents constitute a marginalized, medically complex, and underresearched group who are in high risk of experiencing preventable medication-related harm [3]. Worldwide, older adults in need of specialized health care delivery through primary care, including nursing home care, is expected to increase [2,4]. In terms of improving medication safety in primary health care settings, focus on the most dangerous aspects of systems and the people who are most at risk have been suggested [5]. Highest rates of preventable medication harm have been found in older adult patient care settings [6]. Moreover, nursing homes provide health care for the older adults who are most at risk of experiencing risk factors of medication errors [3,7-9]. In 2021, an 11% increase in adverse events, with a total of 326.416 adverse events, was reported by the Danish Patient Safety Authority in Denmark, and approximately 60% of the events were related to medication errors. A substantial proportion of this increase is reported from municipality health care settings, including nursing homes. This is supported by international findings regarding medication errors in European nursing homes and community care context [7,8,10]. Thus, focus on the interventions to improve medication safety of older adults living in nursing homes is needed. Moreover, multiple work system factors, including persons (resident and staff), organization, tools and technology, tasks, and environment, have been found to affect medication safety in nursing homes. One of the organizational factors, namely, interprofessional collaboration was reported as a relevant factor [11]. These findings reflect recent suggestions for taking a systems approach with focus on organizational issues in terms of minimizing mistakes related to the medication management process [12]. Managing medication of nursing home residents is a highly complex task, often implicating a relatively invisible, multidisciplinary, and cross-sectoral team of health care professionals, providers, as well as the residents and their relatives [13]. However, existing literature remain sparse, heterogenous, and inconclusive when it comes to interventions to improve medication safety in nursing homes [14].

#### Complexity of Context in Primary Care—Toward a Safety II Theoretical Perspective

Absence of accidents and incidents is often referred to as *safety*, which is, ensuring that things do not go wrong. The theoretical underpinning can be defined as Safety I theory, which has been the primary driver of safety improvement efforts globally [15]. Nevertheless, as primary health care constitutes a complex system structure, safety cannot be hypothesized to be achieved through measuring errors to avoid them from reoccurring. The human ability to adapt to varying conditions, thereby increasing resilience within health care systems, cannot be overruled. In theoretical terms this can be defined as Safety II theory, where understanding why things go right most of the time becomes the primary investigatory question to answer [15]. Safety II theory has recently been suggested as a guidance to avoid harm in health care [16], thus enabling prevention of errors through determining underlying mechanisms and resources already in play before errors happen and improving patient safety through no-blame cultures in nursing homes [17]. However, there are no specific evidence-based guidelines on how to realize such safety culture [18,19]. Knowledge on the effects of quality improvement initiatives and interventions to improve medication safety in primary care is based on sparse, highly heterogenous evidence [20,21]. In Denmark, nursing homes are under the responsibility of municipalities who constitute an important part of primary health care. The nursing home setting is highly complex, being both a private home and a center of health care delivery for the residents.

#### Interventions to Improve Medication Safety in Nursing Homes

Development and evaluation of interventions fit to the complex context of nursing homes in large-sample, longitudinal studies have been suggested beneficial to obtain positive, long-lasting effects on medication safety [22]. Within hospital settings, multifaceted approaches and inclusion of stakeholders has been found to be associated with a decrease in medication errors and a positive change in patients’ satisfaction [23]. This supports earlier studies arguing that future interventions targeting medication safety could benefit from being based on multidisciplinary teams focusing on quality as a shared commitment; being multifaceted; and involving all stakeholders, including residents and their relatives [24,25]. Nevertheless, findings are contradictory. A recent study investigating a multifaceted intervention to improve medication safety of nursing home residents did not find positive effects on the health status of nursing home residents [22], contrary to a study aimed to improve medication safety in a hospital setting through a multifaceted interventional strategy [23]. Overall, studies on interventions to improve medication safety in residential health care for older adults are heterogenous in terms of methodologies with inconclusive results [14]. This corresponds to findings of a review on the interventions to optimize medication use within nursing homes [26].

Thus, medication errors and unsafe medication practices continue to concern health care systems worldwide, including nursing home settings. However, earlier research clearly reflects a gap in the knowledge about interventions to improve medication safety in nursing home settings. Patient safety culture is acknowledged as a key to patient safety, both in terms of improvement and effect evaluation [16,27-29] and has been associated with medication errors, also showing improvement potential [19,30]. In 2021, a guiding report on avoiding harm in health care added the importance of focus on primary health care settings, involvement of stakeholders, and cocreation to conquer the challenge of patient safety, including medication safety [16]. Moreover, safety cultures were recommended to be integrated within research designs and as for efforts within health care organizations [16]. This is in alignment with the most recent health work paper by the Organization for Economic Cooperation and Development stating patient safety culture as a recognized factor of importance at all levels of health care. Moroever, instruments to measure patient safety cultures are already in use in 20 of 24 member countries [20].

#### Patient Safety Culture

Patient safety culture is a complex phenomenon that is thought as a subclass of organizational cultures referring to the safety culture related to patients [21]. It is defined as “the values shared among organization members about what is important, their beliefs about how things operate in the organization, and the interaction of these with work unit and organizational structures and systems, which together produce behavioral norms in the organization that promote safety” [31]. Instruments that aim to capture reflections of patient safety cultures have been developed and validated [20,32], with one of the most widely translated and adapted being the “Safety Attitudes Questionnaire” (SAQ), originally developed in the United States in 2006 [33]. The SAQ can be used with dualistic perspectives both to compare different health care employees perceptions and to monitor changes over time [32]. In addition, it allows for comparison between large numbers of sites across settings [33]. Although evidence is sparse and contradictory [34], there is evidence indicating that SAQ is a proper proxy measure of patient safety [35]. SAQ scores have been reported to be directly associated with patient outcomes [32]. The SAQ has been adapted and validated for use in both secondary care and primary care settings [36-40], including Western European nursing homes [38,41] and Danish hospital settings [36].

### Prior Work

International studies have found lower measures of patient safety culture in nursing homes compared with hospital settings [42,43]. Importantly, no single instrument can capture the complexity of safety culture [44], which is regarded a multilayered construct. Outermost layers are considered to be visible and measurable (layers 1 and 2), whereas the deepest layer holding the essence of a culture is measurable only through qualitative means (layer 3) [45]. Layer 3 has not been reflected by earlier research [46], resulting in evidence on patient safety culture in nursing homes being mainly based on quantitative studies. This gap in knowledge could weaken medication safety improvement initiatives [45] and implies a need for in-depth exploration of patient safety culture, including qualitative methods [44-46]. An integrative mixed methods approach to analysis could further strengthen results to gain comprehensive understanding of patient safety culture as a target of intervention to improve medication safety [47].

### Study Objective and Research Questions

Therefore, the primary objective of the Safe Medication in Nursing Home Residents (SAME) study is to develop and evaluate an intervention to improve medication safety of nursing home residents through patient safety culture. First, we aim to develop an intervention to improve medication safety of nursing home residents in a cocreative process, including (1) exploration of patient safety culture as perceived at the frontline of nursing homes (stage 1) and (2) development of a knowledge-based intervention in a multidisciplinary panel informed by findings from focus groups interviews exploring patient safety culture (stage 2). Second, we aim to evaluate the intervention in a randomized controlled trial (RCT) set at nursing homes (stage 3), including a register-based study on patient safety–related outcomes in nursing home residents. Finally, we aim to perform an integrative mixed methods analysis on patient safety culture in nursing homes to comprehensively understand patient safety culture as a complex phenomenon and target of medication safety improvement (stage 4). This could potentially drive a new theory on patient safety culture to guide future efforts to improve medication safety of older adults living in nursing homes. Using an integrated knowledge translation (IKT) approach, we aim to enhance knowledge translation from research into practice through generation of partnership-based, meaningful, acceptable, and feasible results to address medication safety in nursing homes, incorporating the voices of nursing home residents and their relatives. As no current golden standard exists for IKT approach in research, and as the existing guiding tool is based on general assumptions toward cocreativity and knowledge creation, we chose to pragmatically use the 8 guiding principles of IKT [48] that was recently developed to guide research on spinal cord injury, but follow general assumptions and definitions concerning cocreation and knowledge generation, to frame the overall research process of the SAME study. In addition, the IKT project proposal worksheet [49] based on the Canadian Institutes of Health Research’s merit review criteria [50] will be used in the secondary objectives, which are to (1) explore and identify patient safety culture perceived by knowledge users at the frontline of nursing homes, including health care providers and receivers, and find out whether it is possible to explicitly describe implicit, core elements of patient safety culture through in-depth qualitative explorative analysis; (2) cocreate an intervention within existing resource frame to increase medication safety in nursing homes by including stakeholders and knowledge users, and it is based on patient safety culture as perceived at the frontline and guided by Safety II theory; (3) explore the impact of cocreativity in the development of an acceptable and feasible complex intervention to improve medication safety through patient safety culture in nursing homes; (4) develop new, comprehensive understanding of patient safety culture as a complex target to improve medication safety within high-risk complex primary care settings through mixed methods integrative analysis including both in-depth qualitative data on patient safety culture and quantitative data on patient safety climate in nursing homes; and (5) describe the impact of the intervention on quantitative health-related outcomes for safety assessment.

## Methods

### Study Design

#### Overview

**Figure 1:**
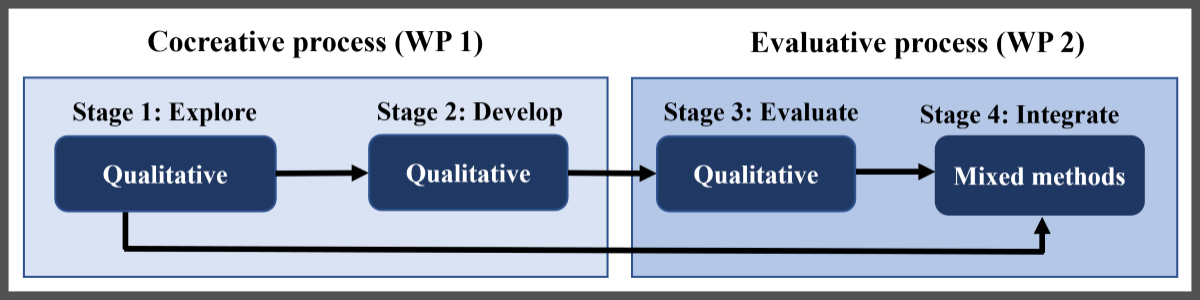
Safe Medication of Nursing Home Residents Through Development and Evaluation of an Intervention overall study design: a fully integrated, mixed methods study following sequential stages covering a cocreative process and a randomized controlled trial to develop and evaluate an intervention to improve medication safety of nursing home residents (black arrows indicate points of data integration). WP: work package.

The SAME study applies an overall theory-informed, fully integrated mixed methods study design that includes an exploratory sequential and IKT-guided research process, integrating 2 work packages including a cocreative intervention developing process (stages 1 and 2) and an evaluative process, including RCT (stage 3) and mixed methods integrative analysis of the intervention (stage 3 and 4). Integration will occur at design, method, and reporting levels to reach comprehensive understanding of patient safety culture as a target to improve medication safety. The SAME study aims to reach partnership-developed results through an IKT approach, thereby increasing the chances of achieving translation of research into practice with a focus on the empowerment of stakeholders, end users, and decision makers to generate useful, meaningful, acceptable, and feasible results. Thus, shifting roles of knowledge users to cocreators will be of primary focus throughout the study (Figure 1). To follow current political recommendations to shift perspective in terms of patient safety, Safety II theory [15] will inform data collection.

#### Setting

Nursing homes registered within the Municipality of Aalborg, The North Denmark Region, Denmark, constitute the main setting of the SAME study. A nursing home is defined as “a facility with a domestic-styled environment that provides 24-hour functional support and care for persons who require assistance with activities of daily living and who often have complex health needs and increased vulnerability” [51].

#### Recruitment

A purposive sampling strategy will be followed through all the stages of SAME, conducted in partnership between the research team and municipal advisory board. The municipal advisory board will actively take part in recruitment through establishing contact with eligible cocreators (stages 1, 2, and 3) and participants (stage 3), including those employed at nursing homes, by sending out information material electronically (email). The research team will be responsible for the recruitment of cocreators not directly employed within municipal settings, including an external consultant in cocreativity and communication, risk managers at hospitals, and volunteers representing nursing home residents and their relatives.

#### Overall Eligibility Criteria

Participants will be eligible for inclusion in the SAME study according to the following criteria: (1) sufficient knowledge of spoken Danish language, (2) being directly or indirectly implicated in the medication management process of nursing home residents, (3) being employed for >2 months at a nursing home registered within the Municipality of Aalborg, and (4) being actively engaged in older adult health care at political or societal level within the Municipality of Aalborg. To represent nursing home residents, we aim to include volunteers from “the senior-council Aalborg” and “The council of elderly” (Table 1); both the groups are characterized by independent, voluntary participation.

**Table 1:** Cocreators of the Safe Medication of Nursing Home Residents Through Development and Evaluation of an Intervention study: subgroups, definitions, and roles.

Subgroup		Definition	Role
**Knowledge users**			
	Municipal management advisory board	Municipal management of the Department of Care for the Elderly and Disabled and Unit of Quality and Innovation	Knowledge on practice Decision makers Feasibility
	Nursing home managers	Nursing home managers and assistants	Knowledge on practice Empowerment Feasibility
	Nursing home care staff	Nursing home care staff (social and health assistants and helpers and home care and nursing home nurses)	Knowledge on practice Empowerment Acceptability Feasibility Scope
	Hospital risk managers	Employed as risk manager at Aalborg University Hospital or employed within the Municipality of Aalborg	Knowledge on practice Feasibility Scope
	General practice dedicated to nursing homes	NH-GP^a^	Knowledge on practice Empowerment Acceptability Feasibility Scope
	Representatives of residents and relatives	Actual relatives of nursing home residents of included nursing homes within the Municipality of Aalborg and representants of “The Senior-council, Aalborg” and “DaneAge Association”	Knowledge on practice Empowerment Acceptability Feasibility Scope
**Stakeholders**			
	Researchers	A multidisciplinary research group will ensure expertise in fields, including qualitative, quantitative, and mixed methods designs in addition to focus on general practice, nursing, and clinical pharmacology	Scientific knowledge Knowledge translation: merging theory and practice
	External consultant in cocreativity and communication	An external expert will play an active part in the research team in the qualitative study stages, from facilitation to data analysis	Knowledge translation: actual practice of cocreativity within municipalities Empowerment of cocreators
	VELUX FONDEN	Noncommercial foundation with grant areas including scientific, environmental, social, and cultural purposes in Denmark and internationally	Funding

^a^NH-GP: Nursing home general practitioners

#### Patient and Public Involvement

We aim to achieve active engagement and empowerment of patients, health care professionals, decision makers and other stakeholders through developing partnerships, with emphasis on equal power but acknowledgment of different roles and responsibility (Table 1). To do so, an overall iterative IKT approach will be applied. IKT is viewed as “an approach or set of processes that can lead to the generation of knowledge for optimizing health care delivery systems and improving health system performance and associated outcomes” [52]. A total of 8 IKT guiding principles are available to support meaningful research, mitigating risk of tokenism. Although relatively recent, the IKT approach has been found useful in other research areas [48] and is in broad terms similar to other collaborative research approaches, including aiming at true partnerships rather than simple engagement; focusing on essential components and processes rather than labels; enabling collaborative research orientations rather than research methods, core values, and principles; and extensive time and financial investment [53]. Thus, in the SAME study, we aim to shift participants and researchers to the role of cocreators following an IKT approach. For practical implication, cocreators include the term “knowledge user” referring to “…an individual who is likely to be able to use the knowledge generated through research to make informed decisions about health policies, programs and/or practices” and “stakeholders” as “individuals, groups or organisations with shared interest in the research; may be in the geographic locality of the research setting or it may be affected by the environmental effects of the research but may not necessarily use the generated knowledge” [54]. To describe and evaluate the level of participation, we will use the International Association for Public Participation Spectrum of Public Participation [55].

Thus, we aim to develop an intervention that will improve medication safety of the older adults with frailty living in nursing homes, and it will be based on coknowledge, generating results perceived as meaningful, useful, acceptable, and feasible by those affected by the research to inform clinical practice. This could help support the ongoing development of sustainable, accessible, and equitable health care systems [56], making room for positive change.

#### SAME Study Cocreators

Table 1 shows an overall presentation of participants in the SAME study. We aim to achieve equally powered partnerships between participants and researchers, shifting participants and researchers into the role of cocreators [53] divided into subgroups following the SAME study IKT approach [57].

### Work Package 1: A Developing Cocreative Process

To reach the overall aim of the SAME study, an intervention to improve medication safety of nursing home residents is aimed in a 2-staged cocreative process (Figure 1). Safety II theory will be used to obtain recommended shift in perspectives in facing health care issues [15,16]. The overall IKT approach of the SAME study will guide the development of cocreators. (Table 1).

#### Stage 1: Qualitative Exploration of Patient Safety Culture in Nursing Homes

The primary objective of stage 1 is to explore patient safety culture in nursing homes, with the perspectives of frontline health care providers being the primary focus. Experienced knowledge will inform the development of the intervention (stage 2) collected through semistructured focus group interviews [58,59]. In addition, field notes will be used as a data source. The medication management process of nursing home residents will be integrated in the development of the semistructured interview guide to ensure the development through medication safety lens of the SAME study.

##### Semistructured Focus Group Interviews

Focus group interviews can be used to gain in-depth understanding of a topic and to identify group norms and cultural values and minimize risk of discrimination regarding reading and writing abilities. Thus, using focus groups to explore patient safety culture could be beneficial with the aim of reaching in-depth knowledge, which is not achieved through quantitative methods [58-60]. All focus group interviews will be facilitated by the external consultant in cocreativity and communication (Table 1). In addition, a researcher (Table 1) will attend to observe and write field notes (Table 1). The first focus group interview will be set to ensure space for the needed adjustments before proceeding with data collection. The municipal risk manager (Table 1) will be invited as an observant of this interview and for a short reflection session at the end of each of the following interviews of approximately 15 minutes.

##### Semistructured Interview Guide

A semistructured interview guide will be developed in collaboration with the research group and an external partner in cocreativity and communication (Table 1). The latter will focus on presentability and general structure of the interview guide, whereas the researchers will account for theoretical information–based and evidence-based outline. To reach in-depth results, 9 intangible domains of organizational culture defined in a recent umbrella review on patient safety culture [44] will inform the interview guide development that will be structured based on Schein’s model of safety culture [45]. Duration of each focus group will be ≤3 hours. This decision was made to face medication safety as a risk area with an expected need for slowly developing a “safe space” for knowledge users not expected to be formerly involved in similar projects, increasing the chance of reaching in-depth results.

##### Study Sample and Size

To help ensure that subgroup norms do not affect data generation negatively and allowing for intimacy, we aim to include a minimum of 3 and ≤5 participants in each focus group. Focus groups will therefore be formed by defined professional subgroups. We aim to include 4 to 6 focus groups, including social and health care assistants, social and health care helpers, general practitioners dedicated to nursing homes, home care nurse and nursing home nurses, and representatives of nursing home residents and their relatives (Table 1). Focus on organizational subgroups could minimize potential negative effects of possible existing hierarchical structures.

##### Outcomes

The primary outcome of stage 1 will be qualitative themes.

##### Data Analysis

Qualitative data will be both field notes and audio recordings that will be transcribed and stored according to current law. Themes will be generated following a triangulated in-person process including researchers with expertise in both qualitative and quantitative methods as well as a representant of end users in the sense of the external consultant in cocreativity and communication, representing cocreators of the SAME study (Table 1). Data analysis will be done following the principles of analyzing in the present [61]. Further validation of the generated themes will be sought through individual triangulation by including researchers representing relevant clinical functions and expertise, including a pharmacist, a nurse, a general practitioner, a qualitative expert, and a quantitative expert.

#### Stage 2: Development of a Knowledge-Based Intervention

In stage 2, the design of the new intervention will be formed. This will be done in a 2-phased process embedded a co-designing process. First, we aim to develop idea generation in a multidisciplinary panel representing a systems view in terms of the medication management process of nursing home residents, and the panel includes nursing home residents and their relatives as well as decision makers. Second, triangulation of ideas will be done with focus on research knowledge. To meet the possibility of using an inadequate number of cocreators to reflect the entire system, the cocreative process will include the investigation of acceptance and usefulness of the intervention through semistructured qualitative focus group interviews with knowledge users and stakeholders in stage 3. The municipal advisory board will play an essential role in stage 2, acting not only as partners in idea generation but also as decision makers in relation to the final intervention design.

##### Data Sources—Collection and Analysis

Data will be qualitative and collected as electronic recordings. Also, a collaborative field note worksheet will be written by facilitators after the workshop. Data analysis will be informed pragmatically by the Delphi method and through a process of data triangulation including stakeholders.

##### Phase 1: Multidisciplinary Panel Workshop

In this phase, we aim to generate ideas based on shared knowledge as an innovative base and to include systems perspective. Experiential knowledge on patient safety culture perceived by nursing home care staff, nursing home residents, and their relatives (Table 1; stage 1, Figure 1) will inform this phase. This will ensure the presence of voices of all knowledge users and minimize potential negative effects of power hierarchical structures. We will ensure acceptable, useful, and meaningful consensus through the presentation of the experiential knowledge from stage 1 as paradoxes by integrating multiple views and possible heterogeneity in the cocreated process. The workshop will be facilitated by the external consultant in cocreativity and communication and 3 representants of the research group (Table 1). The workshop will run over a full working day.

##### Study Sample and Size

A purposive sampling strategy to recruit a multidisciplinary, cross-sectoral panel consisting of knowledge users (Table 1) will be used. This will visualize the relatively invisible team who function in everyday practice and who are implicated in the medication management process of nursing home residents across professions, departments, and health care sectors, including the following:

1. Social assistants and health assistants (n=2)
2. Social helpers and health helpers (n=2)
3. Municipal management (n=2)
4. Nursing home management (n=2)
5. Nursing home general practitioners (n=2)
6. Home care nurses and nursing home nurses (n=2)
7. Risk managers employed at hospital and by the municipality (n=2)
8. Relatives of nursing home residents (n=2)
9. Representants of nursing home residents (“DaneAge association” and “Senior-council, Denmark”; n=2)

##### Outcome, Data Sources, and Collection

The primary outcome of phase 1 will be generation of ideas to improve medication safety, focusing on shared knowledge. A part of a cocreative process, data collection methods have not been predefined but will be developed as part of the research process. The external consultant in cocreativity and communication (Table 1) will play a key role. Both electronic records and field notes will be used as data sources. Results will inform phase 2.

##### Phase 2: The Co-designing Process—Data Sources, Collection, and Analysis

Data will be qualitative and collected as electronic recordings. In addition, a collaborative field note worksheet will be written by facilitators during and after the workshop. Data analysis will be done in a process of data triangulation including relevant stakeholders.

First, a preliminary intervention will be co-designed based on ideas generated at the workshop. Qualitative data will be analyzed guided by “analyzing in the present” [61] based on a triangulated process between participating facilitators after the workshop, following the cocreative approach including the external consultant in cocreativity and communication (Table 1).

Second, a co-designing phase including researchers and decision makers (Table 1) will be done to undertake an organizational and contextualized perspective. This will increase feasibility and acceptability potential of the final intervention design to be evaluated in the RCT (stage 3). Purposely sampled cocreators will be consulted in person through the presentation of the preliminary intervention, thereby leaving room for revision and correction. We aim to include cocreators having expertise in general practice, nursing, and pharmacology, in addition to those from qualitative and quantitative research fields in this phase.

Third, the municipal advisory board will be consulted, as their knowledge concerning organizational factors implicating the intervention will be essential to ensure a local contextualized, final intervention design.

Finally, the intervention will be designed by the research team in terms of explicating the decisions made by all the cocreators. The final intervention will be presented to the municipal advisory board, minimizing the risk of misinterpreted information and increasing the potential for adaptability through management support.

### Work Package 2: Evaluation of Intervention and Understanding of Patient Safety Culture

#### Stage 3: Quantitative Evaluation of the New Intervention in an RCT

In stage 3, we will evaluate the new intervention in a randomized controlled study set at nursing homes. Data on primary outcome will be collected at baseline before rollout of the intervention and at 6-month to 9-month follow-up according to intervention rollout at individual participating nursing homes. Data on secondary outcomes will be collected during the intervention period.

##### Study Sample and Size

A total of 36 public nursing homes were registered within the Municipality of Aalborg in 2021. We expect to recruit a minimum of 22 nursing homes to participate in this phase. Inclusion criteria are the following: to be eligible for inclusion within this study, nursing homes must be supported by a general practitioner registered by the Municipality of Aalborg. Exclusion criteria are the following: if a conflict appears, for example, in relation to other initiatives targeting medication, the Municipality will ensure exclusion of specific nursing homes. Nursing home care staff will be included according to the overall eligibility criteria and definitions defined in Table 1.

As no other studies have investigated the effect of a complex intervention in a mixed methods study design in primary care on SAQ, the sample size cannot be based exclusively on previous study outcomes. We aim to recruit as many respondents as possible from the included nursing homes.

Based on previous studies focusing on related health care setting and culture, we expect mean scale scores of SAQ of 32 to 75 [62,63], and we expect to find an increase of at least 4.0. We expect a lower SD than that reported in previous studies, as these studies have investigated more variable groups of professions. The overall SD (all domains) in home care services have been reported as 9.8 [62]. Thus, with a power of 0.8, significance level (α) of .05, and an expected SD of 9.8, it has been estimated that 192 respondents (96 respondents in the intervention group and 96 respondents in the control group) will be sufficient.

##### Outcomes

The initial problem statement that emerged from the Municipality of Aalborg was that adverse events were increasingly reported, yielding a legally bound challenge that is not yet overcome. Thus, the Municipality sought help for lowering the number of medication errors and unsafe medication practices through collaboration. Although reporting adverse events is an intended goal in safety terms in Denmark, the number of adverse events is not a proper estimate of medication safety.

As a proxy for the overall medication safety, including medication errors, the SAQ has been suggested. A Danish reliable and valid version is available (Danish Safety Attitudes Questionnaire, SAQ-DK). An increase in SAQ-DK would indicate a decrease in medication errors, whereas a decrease would indicate a worsening. We are aware of using the SAQ-DK as the primary outcome with precaution, as the intended increase in reporting adverse events within primary care is still an ongoing issue. However, to the best of our knowledge, no other more suitable measure is available, and therefore, we chose the SAQ as the most reliable and relevant proxy for medication safety in the SAME study.

The primary outcome will be focused on the nursing home frontline, including nursing home care staff’s and nursing home managers’ (Table 1) self-reported perceptions of patient safety culture measured as mean scale SAQ-DK score [36]. The SAQ-DK will be adapted for use in Danish primary health care settings, including nursing homes following the cocreative approach of the SAME study.

Secondary outcomes will include the following: qualitative—perceptions of patient safety culture of relevant cocreators collected through semistructured focus group interviews or individual interviews, depending on the results of the research process; quantitative—register data; that is, sociodemographic data and data on health-related outcomes will be collected at baseline and at follow-up. Data will be collected from relevant Danish registries.

##### Statistical Analysis

An evaluation of the new intervention will be done through comparison of primary outcomes and secondary outcomes between the intervention group and control group of the trial at baseline and 12-month follow-up. Results will be presented as SAQ-DK mean scale scores and SD, using Poisson regression statistical modeling.

*Qualitative data* will be transcribed and stored according to the current law. A structured codebook will be generated to guide thematical analysis [64]. Analysis will be performed by a junior researcher triangulating findings with a senior researcher with expertise in the qualitative research field, using NVivo (QSR International) [65].

*Quantitative data* will be registered and stored in REDCap [66] subsequently being transferred for analyses in Stata (StataCorp).

##### Safety Assessments and Effects

Patient safety–related outcomes will be analyzed through quantitative data collected through national registers; these data include (1) medication (number and type); (2) contacts to a health care system (hospital, nursing home general practitioner, and out-of-hour general practitioner); (3) diagnoses (somatic and psychiatric 10th revision of the International Statistical Classification of Diseases diagnoses); and (4) mortality. Descriptive statistics will be used to compare groups.

#### Stage 4: Mixed Methods Integrative Analysis of Patient Safety Culture: Mixed Methods Analysis

Through a mixed methods integrative analysis, the SAME study aims to gain comprehensive understanding of patient safety culture as a multilayered construct and target of medication safety intervention (stage 4) via different data sets, including (1) a quantitative questionnaire on nursing home care staff and nursing home management’s (Table 1) self-reported perceptions of patient safety climate (SAQ-DK), (2) qualitative data on patient safety culture, and (3) qualitative data from individual interviews with knowledge users. The different sets of data will be collected and analyzed separately for final integration through use of “joint display of data” [67] in a mixed methods analysis. Results could lead to the development of a new theory on patient safety cultures in Danish nursing homes, informing future research and clinical practice in the area.

### Ethics Approval

The North Denmark Region Committee on Health Research Ethics has reviewed and deemed the SAME study exempt according to the study design and the emphasis on the sole use of survey, interview, and national register methodology (2020-000992). The study was registered at and approved by the institutional data protection department, Department of Research Data and Statistics, Aalborg University Hospital (2021-015) and in ClinicalTrials.gov (NCT04990986). Participation will be voluntary, and informed consent will be obtained and can be withdrawn at any point in time. The SAME study will be conducted according to the Declaration of Helsinki (64th WMA General Assembly, Fortaleza, Brazil, October 2013).

## Results

The SAME study is ongoing. Idea groups and experience groups (stage 1) were carried out from April 2021 to September 2021 and the workshop in September 2021. Baseline SAQ-DK data were collected in January 2022, with expected follow-up in January 2023. We expect to finish data analysis in spring 2024.

## Discussion

### Principal Findings

#### Mixed Methods

To impact decision-making, research could benefit from including multiple ways of understanding complex concepts leading to new understanding [68]. Thus, study designs combining mixed methods with participatory approaches could improve research outcome with increasing complexity of the area under investigation, allowing for a deeper understanding of complex phenomena [69-72]. Although sparse, mixed methods studies have been reported, underpinning the benefits in the development and evaluation of interventions [73-75]. Community-based organizations are important stakeholders in health systems, often called upon to use research evidence to inform health care delivery [76]. Consulting key stakeholders and service users, therefore, seems crucial to allow for evidence that is “probably more realistic, acceptable and likely to produce more change” [68]. Combining mixed methods with participatory research approach can help the translation of research into practice and may facilitate meaningful, evidence-based change [69]. Moreover, integration can occur at the level of knowledge. Thus, it is expected that integrative mixed methods analysis including in-depth qualitative exploration will provide a more comprehensive understanding of the complex phenomenon of patient safety culture within high-risk primary care settings, minimizing risk of generating misleading results to guide future clinical practice.

#### IKT Approach

IKT can be used to identify a problem and be able to implement the research recommendations through collaborative research processes. This will form partnerships between researchers and service users [57] aiming to produce meaningful, useful, acceptable, and effectful results [52,57,77]. Importantly, perspectives of representants of an entire organization should be accounted for in terms of investigating safety cultures [44]. Patient participation in the medication process has been stated essential in preventing adverse events, including medication errors [78]. This supports the acknowledgment of patient involvement to avoid harm in health care [16]. Moreover, a scoping review on the IKT approach reported by few studies that included patients who are knowledge users as partners, whereas decision makers were highly represented. Thus, it is anticipated that the IKT approach of the SAME study will increase the chance of identifying and acting upon needs and priorities of knowledge users, including decision makers, nursing home care staff, nursing home residents, and their loved ones, who are rarely integrated as active partners within research on patient safety culture.

#### Overall Discussion Resume

The SAME study will provide insight and knowledge to bridge the gap between research and clinical practice on medication safety strategies targeting older adults with frailty, with emphasis on cocreation. Moreover, understanding patient safety culture through an integrative method could lead to new opportunities of using and analyzing results based on instruments already in play. Including the SAQ as a primary outcome will help generate comparable results. This could increase learning potential across national borders, health care sectors, and local health care organizations and departments as a key to meet the global challenge of patient safety [20]. There is a possibility that results generated could guide the development of a novel framework to facilitate cocreation to improve medication safety in nursing homes.

### Limitations

Systems and nursing home organizational–related factors such as political changes, time schedules, and resources could be limitations in all stages of the SAME study. Moreover, engagement in a knowledge user-researcher partnership is complex, nonlinear, and changeable, presenting a limitation of this study in terms of generalizability, transparency, and feasibility.

### Conclusions

With growing aging populations, there is an increasing scope to analyze medication safety as one of the challenges facing patient safety. The SAME study will help generate evidence on patient safety culture that could help inform and guide future improvement efforts within primary care settings within both political and scientifical scopes.

## Abbreviations

IKT: integrated knowledge translation
RCT: randomized controlled trial
SAME: SAfe MEdication in nursing home residents
SAQ: Safety Attitudes Questionnaire
SAQ-DK: Danish Safety Attitudes Questionnaire

## Data Availability

Data sharing not applicable to this study, as no data sets were yet analyzed during this study.
